# Complete Genome Sequences, Derived by Next-Generation Sequencing, of JC Polyomavirus Strains Isolated from Vietnamese Renal Transplant Recipients

**DOI:** 10.1128/MRA.01350-19

**Published:** 2020-01-09

**Authors:** Trang Dinh Van, Rebecca J. Rockett, An Phan Hai Ha, Trung Vu Nguyen, Dominic E. Dwyer

**Affiliations:** a Clinical Laboratory, National Hospital of Tropical Diseases, Hanoi, Vietnam; b Sydney Medical School, The University of Sydney, Camperdown, NSW, Australia; c Centre for Infectious Diseases and Microbiology Laboratory Services, NSW Health Pathology-Institute of Clinical Pathology and Medical Research, Westmead, NSW, Australia; d International Cooperation Department, Hanoi Medical University, Hanoi, Vietnam; e Kidney Diseases and Dialysis Department, Viet Duc Hospital, Hanoi, Vietnam; f Medical Microbiology Department, Hanoi Medical University, Hanoi, Vietnam; University of Rochester School of Medicine and Dentistry

## Abstract

JC polyomavirus (JCPyV) may cause clinical syndromes such as progressive multifocal leukoencephalopathy in immunocompromised patients. Here, we report seven complete genome sequences of JCPyV genotype 7A, generated directly from urine samples from Vietnamese renal transplant recipients by using rolling-circle amplification and next-generation sequencing.

## ANNOUNCEMENT

JC polyomavirus (JCPyV) is a circular double-stranded DNA virus from the *Betapolyomavirus* genus of the *Polyomaviridae* family. In highly immunosuppressed individuals, JCPyV can cause severe disease, most commonly progressive multifocal leukoencephalopathy (1). JCPyV genotypes have been used to trace human migration, with genotypes 2, 4, and 7 predominating in Southeast Asia (2, 3). This study used a targeted metagenomic method to produce draft full-length JCPyV genome sequences from seven renal transplant recipients from Hanoi, Vietnam.

Seven urine samples were collected from Vietnamese renal transplant recipients between 2015 and 2017. Viral DNA was extracted from 250 μl of urine (already identified as BK polyomavirus positive using the artus BK virus RG PCR kit [Qiagen GmbH, Hilden, Germany]) with the EZ1 DSP virus kit (Qiagen). To ensure sufficient polyomavirus DNA reads from the clinical specimens, a primer-directed rolling-circle amplification method was used to enrich DNA extracts for JCPyV (4). After enrichment, DNA libraries prepared by employing the Nextera XT library preparation kit v2 were sequenced on a NextSeq 500 system (Illumina, Australia) using paired-end 150-bp chemistry.

Raw sequencing reads were trimmed with Trimmomatic v0.36 (sliding window; minimum Phred score, 20) (5). Human reads were removed by mapping reads to a JCPyV reference genome (GenBank accession number J02226) using Burrows-Wheeler alignment (BWA-MEM v0.7.12) (6). Mapped reads were converted to fastq files using SAMtools v1.6 (7) (the numbers of mapped reads [percentage of total reads] are as follows: VN-24, 3,609,902 [99%]; VN-67, 217,170 [52%]; VN-68, 1,346,904 [34%]; VN-214, 5,346,640 [99%]; VN-247, 3,447,166 [99%]; VN-291, 4,586,564 [99%]; and VN-347, 3,557,524 [77%]) (8). Reference mapping, *de novo* assembly, and alignments were conducted using the software package CLC Bio Genomics Workbench v9.0 (Qiagen).

Read mapping to the reference sequence and *de novo* assembly were performed in parallel to confirm the draft genome sequences, because indels and duplications are known to occur within the noncoding control region. Reference mapping resulted in high average coverages (83,802× [VN-24]; 56,675× [VN-67]; 35,023× [VN-68]; 143,065× [VN-214]; 87,133× [VN-247]; 116,532× [VN-291]; and 90,135× [VN-347]) and a GC content of 40.2%. *De novo* assembly produced a single contig spanning the length of the JCPyV genome for specimens VN-24 (96,285×, 5,116 bp), VN-214 (142,644×, 5,115 bp), VN-247 (89,920×, 5,116 bp), and VN-291 (119,699×, 5,116 bp). The remaining specimens produced a maximum of 3 contigs with BLAST+ identity to JCPyV (9) (VN-67, 2 contigs, 53,880×, 3,933 bp; VN-68, 3 contigs, 33,746×, 3,952 bp; VN-347, 2 contigs, 88,362×, 3,937 bp).

**FIG 1:**
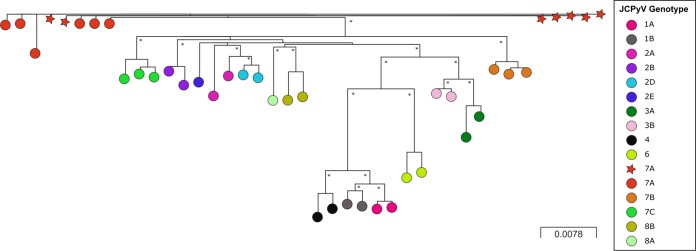
Unrooted phylogenetic tree constructed from the consensus sequences of 43 JCPyV genomes with the noncoding control region removed (alignment length, 4,854 bp). The seven new JCPyV genomes (ENA accession numbers LR215999 to LR216005) produced in this study are depicted as red stars. Previously characterized JCPyV reference sequences (GenBank accession numbers AB038249, U61771, AB048547, AB048550, JVU73501, AF295735, AF295737, AF295738, AF300960, AF300965, AF300963, AF363833, AF363832, AF300950, AF300951, AF300957, AF300959, AF015536, AF004350, AF015533, AF396423, AF295732, AF396432, AF281623, AB038252, AF015537, AF015534, AB038251, AB048576, AB048574, AB048563, J02226, AF281625, and AF015528), representing a range of JCPyV genomes, were also included in the phylogeny (3, 10). The tree was constructed using IQ-TREE software (11) (ModelFinder; substitution model, HKY+F+R2; number of bootstrap replicates, 1,000). The phylogeny was visualized using Microreact and annotated using Inkscape (12). Node colors represent each of the JCPyV genotypes; all of the draft JCPyV genomes produced in this study are genotype 7A. These genomes have high homology (3 to 7 single-nucleotide polymorphisms) to other JCPyV genotype 7A reference sequences (GenBank accession numbers AF295737, AF295738, AF300960, AF300965, AF300963, and U61771) and also are closely related to each other (0 to 3 single-nucleotide polymorphisms) (13). The scale bar represents the number of nucleotide substitutions per site. Asterisks indicate branches with bootstrap support of >85%.

Phylogenetic analysis of draft assemblies indicated that all seven JCPyV genomes were genotype 7A (Fig. 1), which is consistent with reports that this genotype predominates in Southeast Asia. Currently, only a small number of genotype 7 JCPyV genomes are publicly available. This result also demonstrates that JCPyV can be clearly differentiated using metagenomics and that high levels of JCPyV in the viromes of renal transplant recipients may be common.

### Data availability.

Raw fastq files and final consensus draft JCPyV genomes have been deposited in the European Nucleotide Archive (ENA) under accession numbers ERR3561211 to ERR3561215 (fastq files) and LR215999 to LR216005 (genomes).
